# Safety and clinical efficacy of BCMA CAR-T-cell therapy in multiple myeloma

**DOI:** 10.1186/s13045-020-01001-1

**Published:** 2020-12-03

**Authors:** Gils Roex, Marijke Timmers, Kristien Wouters, Diana Campillo-Davo, Donovan Flumens, Wilfried Schroyens, Yiwei Chu, Zwi N. Berneman, Eva Lion, Feifei Luo, Sébastien Anguille

**Affiliations:** 1 Laboratory of Experimental Hematology, Vaccine and Infectious Disease Institute, Faculty of Medicine and Health Sciences, University of Antwerp, Antwerp, Belgium; 2 Division of Hematology and Center for Cell Therapy & Regenerative Medicine, Antwerp University Hospital, Edegem, Belgium; 3 Clinical Trial Center, Antwerp University Hospital, Edegem, Belgium; 4 Biotherapy Research Center, Fudan University, Shanghai, China; 5 Department of Digestive Diseases, Huashan Hospital of Fudan University, Shanghai, China

**Keywords:** BCMA, CAR-T, Multiple myeloma

## Abstract

**Background:**

B-cell maturation antigen (BCMA)-targeted chimeric antigen receptor (CAR)-T-cell therapy is an emerging treatment option for multiple myeloma. The aim of this systematic review and meta-analysis was to determine its safety and clinical activity and to identify factors influencing these outcomes.

**Methods:**

We performed a database search using the terms “BCMA,” “CAR,” and “multiple myeloma” for clinical studies published between 01/01/2015 and 01/01/2020. The methodology is further detailed in PROSPERO (CRD42020125332).

**Results:**

Twenty-three different CAR-T-cell products have been used so far in 640 patients. Cytokine release syndrome was observed in 80.3% (69.0–88.2); 10.5% (6.8–16.0) had neurotoxicity. A higher neurotoxicity rate was reported in studies that included more heavily pretreated patients: 19.1% (13.3–26.7; *I*^2^ = 45%) versus 2.8% (1.3–6.1; *I*^2^ = 0%) (*p* < 0.0001). The pooled overall response rate was 80.5% (73.5–85.9); complete responses (CR) were observed in 44.8% (35.3–54.6). A pooled CR rate of 71.9% (62.8–79.6; *I*^2^ = 0%) was noted in studies using alpaca/llama-based constructs, whereas it was only 18.0% (6.5–41.1; *I*^2^ = 67%) in studies that used retroviral vectors for CAR transduction. Median progression-free survival (PFS) was 12.2 (11.4–17.4) months, which compared favorably to the expected PFS of 1.9 (1.5–3.7) months (HR 0.14; *p* < 0.0001).

**Conclusions:**

Although considerable toxicity was observed, BCMA-targeted CAR-T-cell therapy is highly efficacious even in advanced multiple myeloma. Subgroup analysis confirmed the anticipated inter-study heterogeneity and identified potential factors contributing to safety and efficacy. The results of this meta-analysis may assist the future design of CAR-T-cell studies and lead to optimized BCMA CAR-T-cell products.

## Introduction

Multiple myeloma (MM) is defined by a malignant proliferation of plasma cells in the bone marrow (BM) [1, 2]. As the second most common hematological malignancy after lymphomas, it accounts for 1% of all cancers [3]. Recent epidemiological studies have indicated a steady increase in the incidence and prevalence of MM, mainly attributable to the aging population and therapeutic advances improving survival [4]. Indeed, over the past two decades, the landscape of myeloma treatment has dramatically changed with the advent of several novel therapies, including monoclonal antibodies (mAbs) [5].

**Fig. 1:**
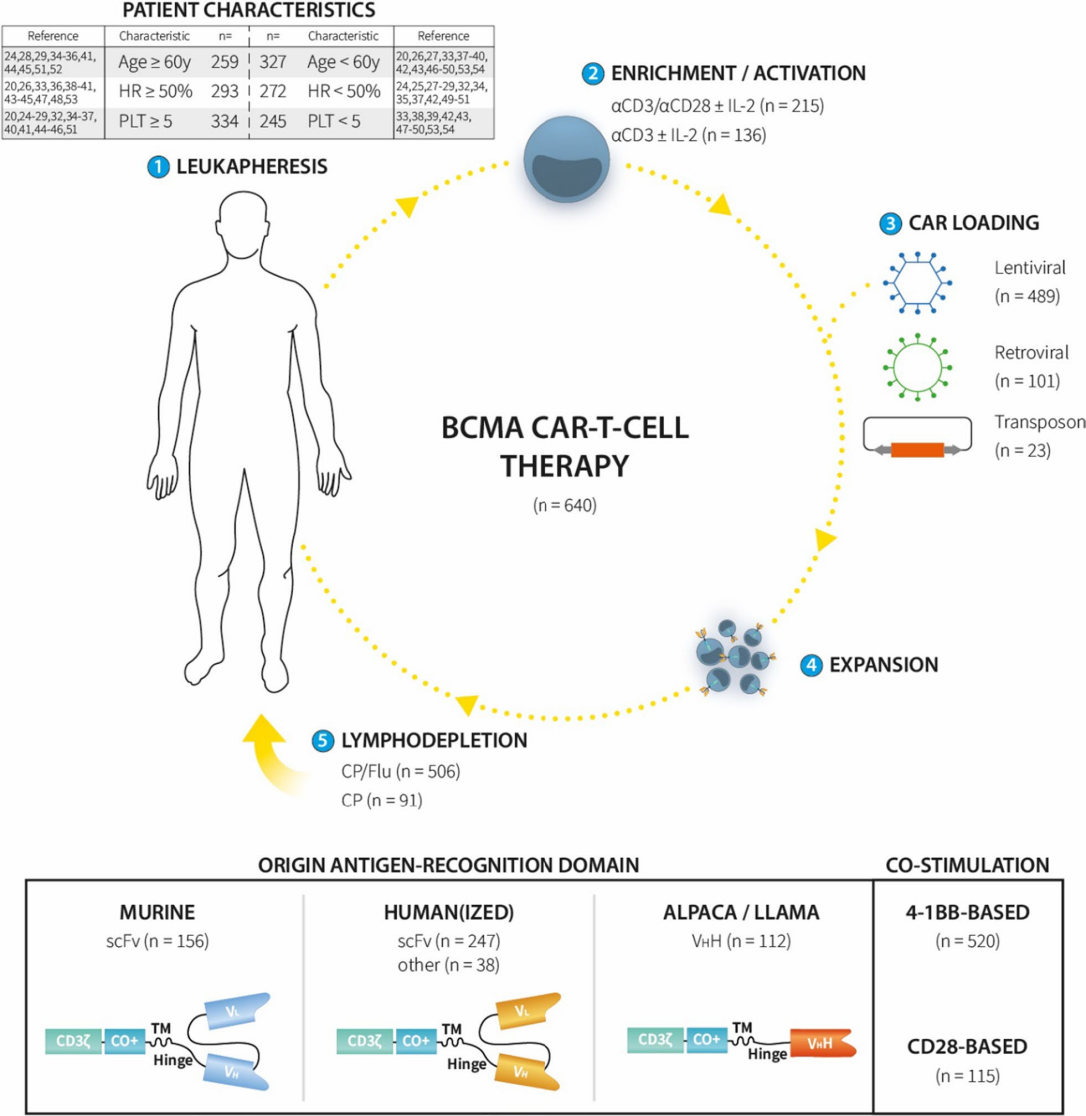
Overview of BCMA CAR-T-cell therapies used to date in multiple myeloma (MM) patients. Twenty-three different BCMA CAR-T-cell products involving 640 patients were identified. All products were derived from autologous T cells collected by apheresis (1), and enriched and activated ex vivo by anti-CD3/CD28 stimulation ± interleukin (IL)-2 or by single anti-CD3 stimulation ± IL-2 (2). The CAR gene was introduced in the T cells by lentiviral or retroviral transduction, or using a transposon (3). The resultant CAR-T cells were then further expanded (4) and administered to the patient by intravenous infusion, usually after lymphodepleting conditioning with cyclophosphamide (CP) ± fludarabine (Flu) (5). The BCMA CAR-T-cell products used to date can be divided into three groups based on the origin of the extracellular antigen-recognition domain: murine, human(ized), or alpaca/llama. The murine and human(ized) CAR constructs are usually based on the antigen-binding domain of a monoclonal antibody (mAb) in single-chain fragment variable (scFv) format with the variable regions of the heavy (*V*_H_) and light chains (*V*_L_) linked together in a single chain. Alpaca/llama BCMA CAR constructs are based on the structure of a camelid nanobody containing one or more V_H_H domains. In addition, the intracellular co-stimulatory domain allows a further subdivision in 4-1BB-based and CD28-based BCMA CAR-T-cell products. Age = studies in whom the median patient age was ≥ or < 60 years. CO+ = co-stimulatory domain. HR = studies with a median of ≥ or < 50% high-risk myeloma patients (based on cytogenetics and/or International Staging System [ISS] score). n = number of patients. PLT = studies in which the median number of prior lines of therapy was ≥ or < 5. TM = transmembrane domain.

Recently, chimeric antigen receptor (CAR)-T-cell immunotherapy has entered the clinical trial arena [6, 7]. CAR-T cells are autologous lymphocytes collected by leukapheresis and genetically modified (most often by lentiviral or retroviral transduction) to express a CAR. Following ex vivo expansion, the cells are then reinfused to the patient who is usually first conditioned with lymphodepleting chemotherapy (Fig. 1) [8]. CARs are synthetic receptors that bear characteristics of a mAb and a T-cell receptor (TCR); they contain an antigen-recognition domain from a mAb (usually in single-chain variable fragment [scFv] format) and CD3ζ [9]. The mAb part is responsible for HLA-independent binding of the CAR-T cell to a target expressed on the tumor cell surface, whereas the CD3ζ chain triggers T-cell activation by mimicking TCR signaling. Most CAR constructs also contain one (2nd generation) or more (3^rd^ generation) co-stimulatory domains, such as 4-1BB or CD28 (Fig. 1) [8].

Although several antigens are undergoing clinical evaluation, B-cell maturation antigen (BCMA) has been the most popular myeloma target antigen so far [10–12]. BCMA is involved in cell survival and is expressed exclusively on the surface of B-cell lineage cells, including malignant plasma cells [10, 11]. The impressive clinical results of CD19-targeted CAR-T cells in CD19^+^ hematological malignancies [6, 13, 14] have created high expectations for CAR-T-cell therapy in other cancers [15]. However, it remains unclear whether these expectations are justified in the context of MM since doubts have recently been raised about the durability of therapeutic activity [16]. Moreover, CAR-T-cell therapy can produce potentially life-threatening toxicities, such as cytokine release syndrome (CRS) and neurotoxicity [17].

Current evidence on BCMA-targeted CAR-T-cell therapy in MM is restricted to relatively small, non-randomized early phase clinical trials. Hence, at this stage, it is difficult to obtain a clear sight on the toxicity and efficacy that can be expected from this novel therapeutic approach in relapsed/refractory MM patients. To the best of our knowledge, there has been only one attempt so far to systematically aggregate the outcome data of BCMA CAR-T-cell clinical studies [18]. In that report, Gagelmann et al. included 15 studies comprising a total of 285 patients. Here, we were able to identify 27 studies involving 23 different BCMA CAR-T-cell products and a total of 640 patients, making it the most comprehensive systematic review and meta-analysis to date of the safety and clinical efficacy of BCMA-targeted CAR-T-cell therapy in MM. Moreover, this study is also the first to identify potential patient- and treatment-related factors influencing toxicity and efficacy, which helps us to understand the different outcomes between bb2121 (idecabtagene vicleucel) and LCAR-B38M (ciltacabtagene autoleucel), the two most advanced, late-stage BCMA CAR-T-cell products which are likely to receive regulatory approval in the years to come. Furthermore, controlled trials are lacking, making it challenging to assess the true progression-free survival (PFS) benefit that is reported in individual clinical studies. In this meta-analysis, we incorporated a surrogate control arm, composed of patients treated with inactive doses of BCMA CAR-T cells. PFS data from this control population were used to determine the expected outcome in order to more accurately assess the therapeutic benefit of BCMA CAR-T-cell therapy in relapsed/refractory MM patients.

## Methods

### Search strategy and selection criteria

**Fig. 2:**
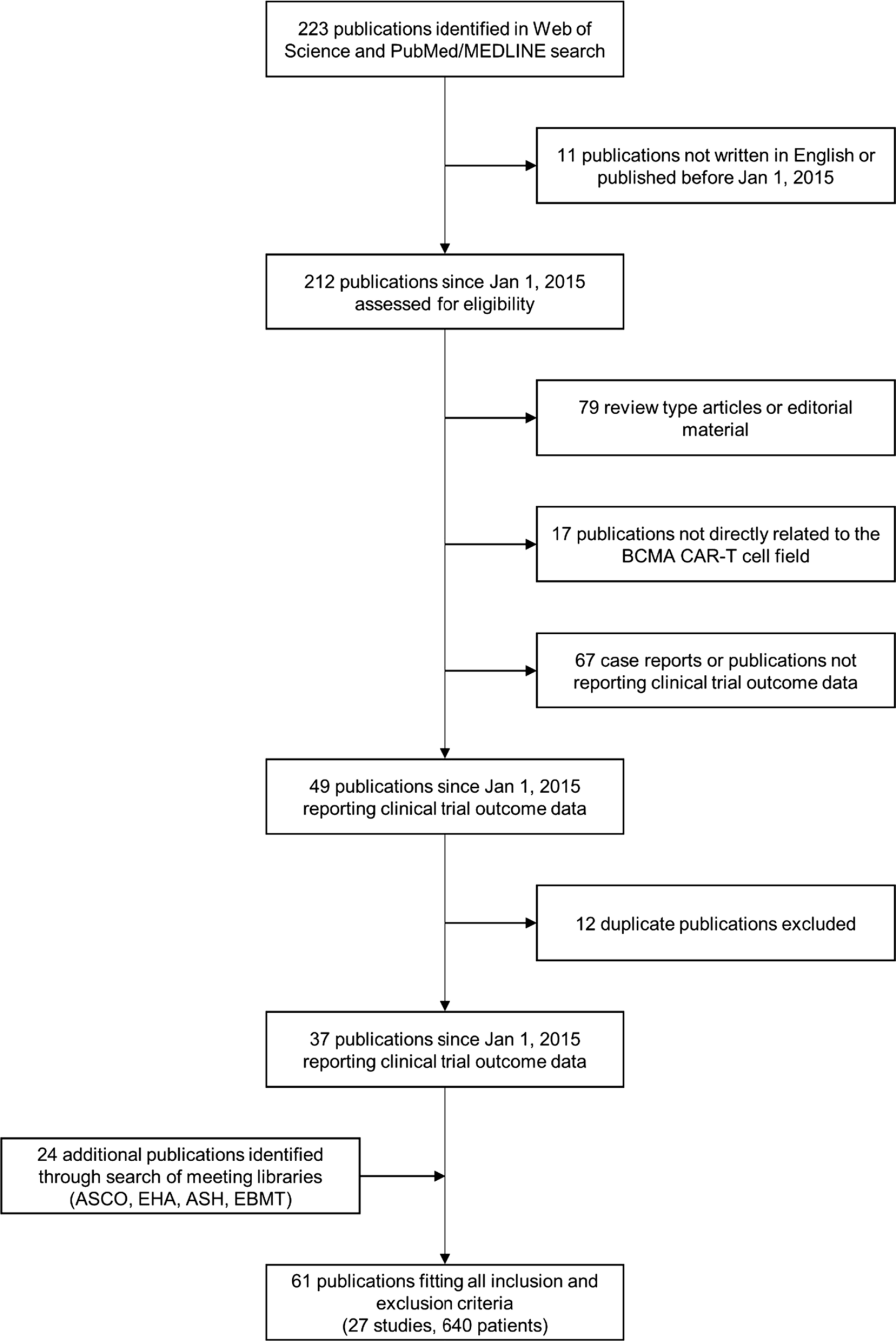
Search strategy and study selection. ASCO = American Society of Clinical Oncology. ASH =American Society of Hematology. BCMA = B-cell maturation antigen. CAR = chimeric antigen receptor. EBMT = European Group for Blood and Marrow Transplantation. EHA = European Hematology Association

This study involves a systematic review and meta-analysis of the risks and benefits of BCMA CAR-T-cell therapy in MM patients. Relevant clinical studies were identified by a systematic search of Web of Science (Clarivate Analytics) and PubMed/MEDLINE using the following search terms: “B-cell maturation antigen” or “BCMA,” “chimeric antigen receptor” or “CAR,” and “multiple myeloma.” Additional records were retrieved by screening published conference abstracts of American Society of Clinical Oncology (ASCO), American Society of Hematology (ASH), European Group for Blood and Marrow Transplantation (EBMT), and European Hematology Association (EHA). All clinical trial designs (i.e., controlled and uncontrolled studies) were considered. Since the first clinical report of CAR-T-cell therapy in MM was published in 2015 [19], the search was restricted to studies published between January 1, 2015, and January 1, 2020. Only clinical trials registered on Clinicaltrials.gov (NCT-number) or Chinese Clinical Trial Registry (ChiCTR-number) and published in English, either as full scientific article or as abstract during the annual scientific meetings of ASCO, ASH, EBMT, or EHA, were taken into consideration. Patient data were solely extracted from these publications, and no requests for additional original patient data were made to the authors of these studies. Reviews and non-scientific publications were not used for data collection to avoid duplicate data, but were used to ensure accurate and appropriate data selection. Database searches and data collection were conducted independently by three authors (GR, MT, and SA). Data were omitted if no unanimous consensus over their inclusion was found. The PRISMA flow diagram in Fig. 2 depicts the search strategy that was followed to identify the relevant publications (Fig. 2).

### Data analysis

Table S1 (Additional file 1) provides an overview of the 61 publications that were retrieved following the PRISMA flow diagram depicted in Fig. 2. Based on the clinical trial registration number (NCT-number or ChiCTR-number), the CAR-T-cell product name, and the study group (lead author and affiliation), we were able to identify 27 different studies comprising 640 unique patients. For several studies, overlapping publications were identified; to avoid duplicate data, only the most recent and/or the largest (based on the number of included patients) records were considered (Additional file 1: Table S1). As shown in Table S1, there were two exceptions to this rule. For study NCT02546167 (CART-BCMA UPenn), we decided to use the full publication [20] rather than the meeting abstract [21]. For study NCT03661554 (BCMA nanoantibody), the latest publication involving 16 CAR-T-infused patients was not considered because outcome data were incompletely reported (only for 7 patients) [22].

Primary outcome measures were CAR-T-cell-related toxicities (i.e., CRS and neurotoxicity), and objective response rate (ORR). ORR was defined as the sum of (stringent) complete responses ([s]CR) and (very good) partial responses ([VG]PR), according to IMWG criteria [23]. Progression-free survival (PFS) was used as secondary outcome measure. We collected data on the following patient- and disease-related variables: number of patients, median age, myeloma risk (based on cytogenetics and/or International Staging System [ISS] score), and prior lines of therapy. Information on the following treatment-related variables was extracted: origin and type of the CAR antigen-recognition domain, enrichment/activation method, loading strategy, type of co-stimulatory domain, cell dosage, and lymphodepletion regimen.

We conducted a meta-analysis for proportions to estimate the overall proportion of CRS/neurotoxicity and ORR/CR. Because of the diversity between the studies, a random-effects model was used. Heterogeneity was judged by forest plots and *I*^2^*.* Results are reported as proportions with 95% confidence interval (CI). Subgroup analyses were performed to assess differences between groups of studies. P values were calculated based on the between subgroups heterogeneity statistic.

Median PFS with 95% CI was calculated from individual patient data, which were retrieved using computerized analysis of published Swimmer plots and/or Kaplan–Meier survival curves. We verified the correctness of the retrieved data by back-checking that the calculated median PFS was identical to the published median PFS of each study. A comparative analysis was performed between CAR-T cells used at active doses with inactive doses, where an inactive dose was defined as a CAR-T cell dose that failed to produce both CRS and ORR rates of > 50%. This corresponded to the patients included in the lowest dose cohorts of the following four early phase BCMA CAR-T-cell studies with a dose-escalation design: NCT02658929 [24], NCT02546167 [20], NCT02215967 [25], and NCT03070327 [26]. In the absence of randomized controlled trials, the latter served as a surrogate control group to determine the expected PFS. A marginal Cox regression model with clustering per study was used to assess differences in PFS between the subgroups. All statistical analyses were performed using R v3.4.4. (R Foundation for Statistical Computing, Vienna, Austria). This study was registered with PROSPERO (CRD42020125332).

## Results

As shown in Table 1 and Figs. 1 and 2, 27 studies involving 23 different BCMA CAR-T-cell products were identified. Data were available from 640 BCMA CAR-T-cell treated patients. For 11 CAR-T-cell products, the extracellular BCMA-recognition domain of the CAR consisted of a human(ized) mAb in scFv format (Table 1) [55]. In one study (NCT03288493), the antigen-recognition domain was composed of a centyrin, a human fibronectin type III-based antibody mimetic [45, 56], while another (NCT03602612) used a human heavy-chain-only binding domain [44]. All other studies used non-human antibodies, either murine scFV mAb or nanobodies derived from alpaca or llama [46, 57]. Bb2121 and LCAR-B38M, the two most advanced BCMA CAR-T-cell products, used a murine- and llama antibody-based CAR construct, respectively (Table 2). The method used for T-cell enrichment/activation was not reported in the majority of the studies; anti-CD3 and anti-CD28 antibodies (usually coupled to magnetic beads) or an anti-CD3 antibody alone, with or without interleukin (IL)-2, were mostly used [58]. Lentiviral (489/640 patients; 76.4%) and, to a lesser extent, gamma-retroviral transduction (101/640 patients; 15.8%) were the preferred transduction methods (Table 1). NCT03288493 (23/640 patients; 3.6%) was the only clinical trial so far in which a non-viral delivery method was applied (i.e., a transposon). In two trials (ChiCTR-1800018143 and ChiCTR-1900027678), the method of CAR loading was not defined (Table 1) [33, 54]. In 520/640 patients (81.3%), a 4-1BB-based second-generation CAR construct was used; the other patients received BCMA CAR-T cells with a CD28 co-stimulatory domain (either alone or in combination with OX40 or 4-1BB). One study (ChiCTR-1900027678) did not disclose the type of co-stimulatory domain [54]. CAR-T cell dosages varied considerably across the different studies, from 0.07 × 10^6^/kg to > 1000 × 10^6^ cells. This variation is also exemplified in Table 2, comparing bb2121 and LCAR-B38M, showing a tenfold difference between both studies in CAR-T-cell dosage used (Table 2). Cyclophosphamide, usually in combination with fludarabine, was the most frequently used lymphodepleting chemotherapy regimen.

**Table 1:** Multiple myeloma CAR-T-cell clinical trials targeting BCMA

Trial # ^ref.^ (product name)	*n* =	Origin mAb	Expansion	Loading	Co-stimulation	T-cell dosage	Conditioning	Toxicity	Clinical response
ChiCTR-OIC17011272 [27] (CD19 & BCMA CAR-T)	21	Murine scFv	aCD3	Lentiviral	4-1BB	1 × 10^6^/kg	CP/Flu	CRS gr. 1–2 (86%), gr. ≥ 3 (5%) Neurotoxicity (10%)	sCR/CR (57%)/VGPR (24%) PR (14%)
NCT02658929 [24, 28] (bb2121)	43/39	Murine scFv	aCD3 + aCD28	Lentiviral	4-1BB	50–800 × 10^6^	CP/Flu	CRS gr. 1–2 (58%), gr. ≥ 3 (5%) Neurotoxicity (33%)	sCR/CR (44%)/VGPR (23%) PR (10%)
NCT03274219 [29] (bb21217)	38	Murine scFv	aCD3 + aCD28 + PI3k inhibitor	Lentiviral	4-1BB	150–450 × 10^6^	CP/Flu	CRS gr. 1–2 (61%), gr. ≥ 3 (5%) Neurotoxicity (24%)	sCR/CR (13%)/VGPR (34%) PR (5%)
ChiCTR-OPC16009113 [30, 31] (BCMA-CAR T)	28	Murine scFv	aCD3	Lentiviral	CD28/4-1BB	5.4–25 × 10^6^/kg	CP/Flu	CRS gr. ≥ 3 (14%)	sCR/CR (61%)/VGPR (4%) PR (21%)
NCT02215967 (1) [25, 32] (NCI BCMA CAR-T)	10	Murine scFv	aCD3 + IL-2	Retroviral	CD28	0.3–3 × 10^6^/kg	CP/Flu	CRS gr. 1–2 (30%)	VGPR (10%) PR (10%)
NCT02215967 (2) [25] (NCI BCMA CAR-T)	16	Murine scFv	aCD3 + IL-2	Retroviral	CD28	9 × 10^6^/kg	CP/Flu	CRS gr. 1–2 (56%), gr. ≥ 3 (38%) Neurotoxicity (6%)	sCR/CR (13%)/VGPR (50%) PR (19%)
ChiCTR-1800018143 [33] (BM38 CAR)	22	Humanized scFv	ND	ND	4-1BB	0.5–4 × 10^6^/kg	CP/Flu	CRS gr. 1–2 (68%), gr. ≥ 3 (23%)	sCR/CR (55%)/VGPR (9%) PR (24%)
NCT02546167 [20] (CART-BCMA UPenn)	25	Human scFv	aCD3/CD28	Lentiviral	4-1BB	50–500 × 10^6^	CP or none	CRS gr. 1–2 (56%), gr. ≥ 3 (32%) Neurotoxicity (32%)	sCR/CR (8%)/VGPR (20%) PR (20%)
NCT03302403, NCT03380039, NCT03716856 [34, 35] (CT053)	24	Human scFv	aCD3/CD28	Lentiviral	4-1BB	50–180 × 10^6^	CP/Flu	CRS gr. 1–2 (63%) Neurotoxicity (8%)	sCR/CR (79%)/VGPR (4%) PR (4%)
NCT03430011 [36] (JCARH125)	44	Human scFv	ND	Lentiviral	4-1BB	50–450 × 10^6^	CP/Flu	CRS gr. 1–2 (70%), gr. ≥ 3 (9%) Neurotoxicity (25%)	sCR/CR (27%)/VGPR (20%) PR (34%)
NCT03815383 [37] (C-CAR088)	5	Human scFv	ND	Lentiviral	4-1BB	1–3 × 10^6^/kg	CP/Flu	CRS gr. 1–2 (80%)	sCR/CR (20%)/VGPR (60%) PR (20%)
ChiCTR-1800018137 [38] (CT103A)	18	Human scFv	ND	Lentiviral	4-1BB	1–6 × 10^6^/kg	CP/Flu	CRS gr. 1–2 (72%), gr. ≥ 3 (22%)	sCR/CR (67%)/VGPR (17%) PR (17%)
NCT03549442 [39] (CART-BCMA + CTL119)	16	Human scFv	ND	Lentiviral	4-1BB	500 × 10^6^	CP/Flu	CRS gr. 1–2 (88%)	sCR/CR (19%)/VGPR (25%) PR (25%)
NCT03338972 [40] (FCARH143)	11	Human scFv	aCD3/CD28	Lentiviral	4-1BB + EGFRt	50–150 × 10^6^	CP/Flu	CRS gr. 1–2 (91%) Neurotoxicity (9%)	sCR/CR (55%)/VGPR (36%) PR (9%)
NCT03502577 [41] (FCARH143 + GSI)	10	Human scFv	ND	Lentiviral	4-1BB + EGFRt	50–300 × 10^6^	CP/Flu	CRS gr. 1–2 (60%), gr. ≥ 3 (40%) Neurotoxicity (60%)	sCR/CR (30%)/VGPR (50%) PR (20%)
NCT03196414 [42] (SZ-MM-CART01)	29/28	Humanized scFv	aCD3	Lentiviral	CD28/OX40	20–82 × 10^6^/kg	CP/Flu	CRS gr. 1–2 (66%), gr. ≥ 3 (34%) Neurotoxicity (3%)	sCR/CR (54%)/VGPR (4%) PR (29%)
NCT03455972 [43] (SZ-MM-CART02)	32	Humanized scFv	aCD3	Lentiviral	CD28/OX40	50 × 10^6^/kg	BUCY or Mel + autoHSCT	CRS gr. 1–2 (97%), gr. ≥ 3 (3%)	sCR/CR (72%)/VGPR (ND) PR (ND)
NCT03070327 [26] (MCARH171)	10/11	Human scFv	ND	Retroviral	4-1BB + EGFRt	1 × 10^6^/kg or 150–450 × 10^6^	CP/Flu or CP	CRS gr. 1–2 (40%), gr. ≥ 3 (20%) Neurotoxicity (10%)	VGPR (45%) PR (18%)
NCT03602612 [44] (FHVH33)	15	Human VH	ND	Retroviral	4-1BB	ND	CP/Flu	CRS gr. 1–2 (87%), gr. ≥ 3 (7%) Neurotoxicity (27%)	sCR/CR (20%)/VGPR (7%) PR (53%)
NCT03288493 [45] (P-BCMA-101)	23/19	Human centyrin	None	Transposon	4-1BB + rimiducid SS	51–1143 × 10^6^	CP/Flu	CRS gr. 1–2 (9%) Neurotoxicity (4%)	sCR/CR + VGPR (26%) PR (42%)
NCT03661554 [46] (BCMA nanoantibody)	9	Alpaca V_H_H	ND	Lentiviral	4-1BB	250–900 × 10^6^	CP/Flu	CRS gr. 1–2 (67%), gr. ≥ 3 (22%) Neurotoxicity (11%)	sCR/CR (56%)/VGPR (33%) PR (11%)
NCT03090659 (1) [47, 48] (LCAR-B38M)	17	Llama V_H_H	aCD3/CD28 + IL-2	Lentiviral	4-1BB	0.21–1,52 × 10^6^/kg	CP/Flu or CP	CRS gr. 1–2 (59%), gr. ≥ 3 (41%)	sCR/CR (82%)/VGPR (6%)
NCT03090659 (2) [49, 50] (LCAR-B38M)	57	Llama V_H_H	aCD3/CD28 + IL-2	Lentiviral	4-1BB	0.07–2,1 × 10^6^/kg	CP	CRS gr. 1–2 (82%), gr. ≥ 3 (7%) Neurotoxicity (2%)	sCR/CR (73%)/VGPR (4%) PR (11%)
NCT03548207 [51] (LCAR-B38M)	29	Llama V_H_H	ND	Lentiviral	4-1BB	0.5–0.9 × 10^6^/kg	CP/Flu	CRS gr. 1–2 (86%), gr. ≥ 3 (7%) Neurotoxicity (10%)	sCR/CR (69%)/VGPR (17%) PR (14%)
ChiCTR-1800017404 [52] (BCMA CAR-T)	33/32	ND	ND	Lentiviral	4-1BB	1–6 × 10^6^/kg	CP/Flu	CRS gr. 1–2 (52%), gr. ≥ 3 (48%)	sCR/CR (66%)/VGPR (22%) PR (13%)
NCT03093168 [53] (HRAIN BCMA-CART)	49	ND	ND	Retroviral	4-1BB + EGFRt	9 × 10^6^/kg	CP/Flu	CRS gr. 1–2 (12%), gr. ≥ 3 (6%)	sCR/CR (45%)/VGPR (18%) PR (14%)
ChiCTR-1900027678 [54] (GC012F)	5	ND	ND	ND	ND	1–2 × 10^6^/kg	CP/Flu or none	CRS gr. 1–2 (80%)	sCR/CR (20%)/VGPR (80%)
**Pooled studies**	**639/630**							**CRS gr. 1–4 (80.3%)**	**ORR (80.5%)**
								**(95% CI 69.0–88.2;* I***^**2**^ **= 83%)**	**(95% CI 73.5–85.9;* I***^**2**^** = 61%**)
								**Neurotoxicity (10.5%)**	
								**(95% CI 6.8–16.0;** ***I***^**2**^ **= 58%)**	

aCD3 + aCD28 = anti-CD3 and anti-CD28 antibodies. aCD3/CD28 + IL-2 = anti-CD3 and anti-CD28-coated beads plus interleukin-2. AutoHSCT = autologous hematopoietic stem cell transplant. BCMA = B-cell maturation antigen. BUCY = busulfan and cyclophosphamide. CAR = chimeric antigen receptor. CP = cyclophosphamide. CR = complete response. CRS = cytokine release syndrome. EGFRt = truncated epidermal growth factor receptor. Flu = fludarabine. Gr. = grade. GSI = gamma-secretase inhibitor. IL-2 = interleukin-2. Mel = melphalan. n = number of patients evaluable for toxicity/clinical response. ND = not disclosed. PI3k = phosphoinositide 3-kinase. PR = partial response. scFv = single-chain fragment variable. SS = safety switch. sCR = stringent complete response. Trial # = study registration number in Clinicaltrials.gov (NCT#) or Chinese Clinical Trial Registry (ChiCTR-#). VGPR = very good partial response. V_H_H = nanobody

**Table 2:** Comparison of KarMMa (bb2121) and LEGEND-2 (LCAR-B38M) clinical studies

	bb2121 / KarMMa [59]	LCAR-B38M / LEGEND-2 (Xi’an site) [49, 50]
Alternative product name	ide-cel	cilta-cel
Trial # (study phase)	NCT03361748 (phase II)	NCT03090659 (phase I)
*n* of patients	128 (54 at RD of 450 × 10^6^)	57
Expansion method	aCD3 + aCD28	aCD3/CD28 + IL-2
Loading method	Lentiviral	Lentiviral
CAR-T structure	Murine scFv	Llama 2xV_H_H
	
Lymphodepletion	CP/Flu	CP
CAR-T cell dosage(s)	150–300 to 450 × 10^6^	32.3 × 10^6^ (3.3 to 126.2 × 10^6^)
Patient characteristics		
Age (range), y	61 (33–78)	54 (27–72)
Median n PLT (range)	6 (3–16)	3 (1–9)
High-risk features^a^	51%	37%
CRS	96.3%^b^	89.5%
Gr. 1–2	90.7%	82.5%
Gr. ≥ 3	5.6%	7.0%
Median onset (range)	1d (1–10)	9d (1–19)
Median duration (range)	7d (1–63)	9d (3–57)
Tocilizumab use	67%	46%
Neurotoxicity	20.4%^b^	1.8%
ORR	82%^b^	88%
MRD^−^ CR	28%	68%
CR	11%	5%
VGPR	26%	4%
PR	17%	11%
Median PFS (95% CI)	12.1m (8.8–12.3)^b^	19.9m (9.6–31)

aCD3 + aCD28 = anti-CD3 and anti-CD28 antibodies. aCD3/CD28 + IL-2 = anti-CD3 and anti-CD28-coated beads plus interleukin-2. cilta-cel = ciltacabtagene autoleucel. CP = cyclophosphamide. CP/Flu = cyclophosphamide plus fludarabine. CR = complete response. CRS = cytokine release syndrome. d = days. Gr. = grade. ide-cel = idecabtagene vicleucel. m = months. MRD = minimal residual disease. n = number. ORR = objective response rate. PFS = progression-free survival. PLT = prior lines of treatment. RD = recommended dose. scFv = single-chain variable fragment. (VG)PR = (very good) partial response. VHH = heavy-chain variable region. Trial # = study registration number in Clinicaltrials.gov (NCT#). y = years

^a^High-risk defined as R-ISS stage 3 and/or high-risk genetics (del(17p), t(4;14), t(14;16))

^b^Data shown for the 450 × 10^6^ dose cohort only

Among 639 patients evaluable for safety, 80.3% (69.0–88.2) experienced CRS (Table 1). CRS is graded on a scale from 1 to 4 [17]; severe CRS (i.e., grade ≥ 3) occurred in 14.1% of patients (9.6–20.4). As shown in Table 2, detailing the key differences between the two most advanced BCMA CAR-T products bb2121 and LCAR-B38M, the median time of CRS onset varied greatly between 1 and 9 days. The median duration was between 7 and 9 days for bb2121 and LCAR-B38M, respectively; CRS could last to up to 2 months (Table 2). The pooled CRS rate was 61.0% (35.3–81.8; *I*^2^ = 84%), 83.8% (70.9–91.7; *I*^2^ = 71%), and 91.0% (83.8–95.2; *I*^2^ = 0%) in studies using CAR constructs with murine-based, human(ized), and alpaca/llama-derived antigen-binding domains, respectively (Additional file 2: Table S2 and Fig. S1). Despite the apparently lower CRS rate in studies using murine scFv-based CAR constructs, individual studies revealed a clear “dose-toxicity” relation. For example, with the bb2121 CAR-T product, which contains a murine anti-BCMA scFv, a CRS rate of 96.3% was noted at the recommended phase II dose of 450 × 10^6^ cells (Table 2), whereas it was only 75.7% and 50.0% at the 300 × 10^6^ and 150 × 10^6^ dose levels, respectively [59].

The pooled neurotoxicity rate was 10.5% (6.8–16.0), with a considerable variation between the different studies. For example, in the bb2121 study, 20.4% of the patient experienced some sort of neurological symptoms, whereas only 1.8% of the LCAR-B38M-treated patients had neurotoxicity (Table 2). The origin of the antigen-recognition domain (murine, human(ized), or alpaca/llama) had no impact on neurotoxicity (Additional file 3: Table S3). Lymphodepletion with cyclophosphamide and fludarabine, a known neurotoxic agent, did not lead to more neurological events as compared to cyclophosphamide alone or no lymphodepletion. A lower rate of neurotoxicity was observed in studies that used anti-CD3 mAbs alone instead of anti-CD3/CD28 mAbs for T-cell enrichment/activation (4.9% [2.1–10.9; *I*^2^ = 0%] versus 15.9% [8.1–28.9; *I*^2^ = 66%]; *p* = 0.028). A similar observation was made for studies that used CD28 instead of 4-1BB as co-stimulatory backbone (3.4% [1.2–9.3; *I*^2^ = 0%] versus 12.9% [8.2–19.6; *I*^2^ = 59%]; *p* = 0.018). A higher rate of neurotoxicity was observed in studies in which the median patient age was ≥ 60 years (20.5% [12.5–31.9; *I*^2^ = 63%] versus 6.4% [3.3–12.0; *I*^2^ = 38%]; *p* = 0.0043), and in studies in which the median number of prior lines of therapy was ≥ 5 (19.1% [13.3–26.7; *I*^2^ = 45%] versus 2.8% [1.3–6.1; *I*^2^ = 0%]; *p* < 0.0001; Additional file 3: Fig. S2).

A total of 630 patients were evaluable for clinical response (Table 1). The pooled ORR was 80.5% (73.5–85.9) with (s)CR in 44.8% (35.3–54.6) of patients. Responses occurred rapidly, usually within the first month after CAR-T-cell infusion. Despite the higher likelihood to achieve a deep response in studies that included less pretreated patients (CR: 57.6% [45.2–69.0; *I*^2^ = 63%]; *p* = 0.011), a (s)CR rate of 32.9% (21.1–47.4; *I*^2^ = 77%) was still achieved in studies with a median of ≥ 5 prior lines of therapy. Concerning the treatment-related variables, a superior CR rate of 71.9% (62.8–79.6; *I*^2^ = 0) was noted in studies with an alpaca/llama-derived BCMA-recognition domain (*p* < 0.0001 compared to their human and murine counterparts; Additional file 4: Fig. S3). Responses were usually deeper in studies that used an alpaca/llama-based anti-BCMA CAR construct, as exemplified by LCAR-B38M in Table 2. Finally, the CR rate was only 18.0% (6.5–41.1; *I*^2^ = 67%) in studies that used a retroviral instead of a lentiviral vector (50.6% [39.8–61.4; *I*^2^ = 77%]) for CAR-T-cell transduction (*p* = 0.015; Additional file 4: Table S4).

**Fig. 3:**
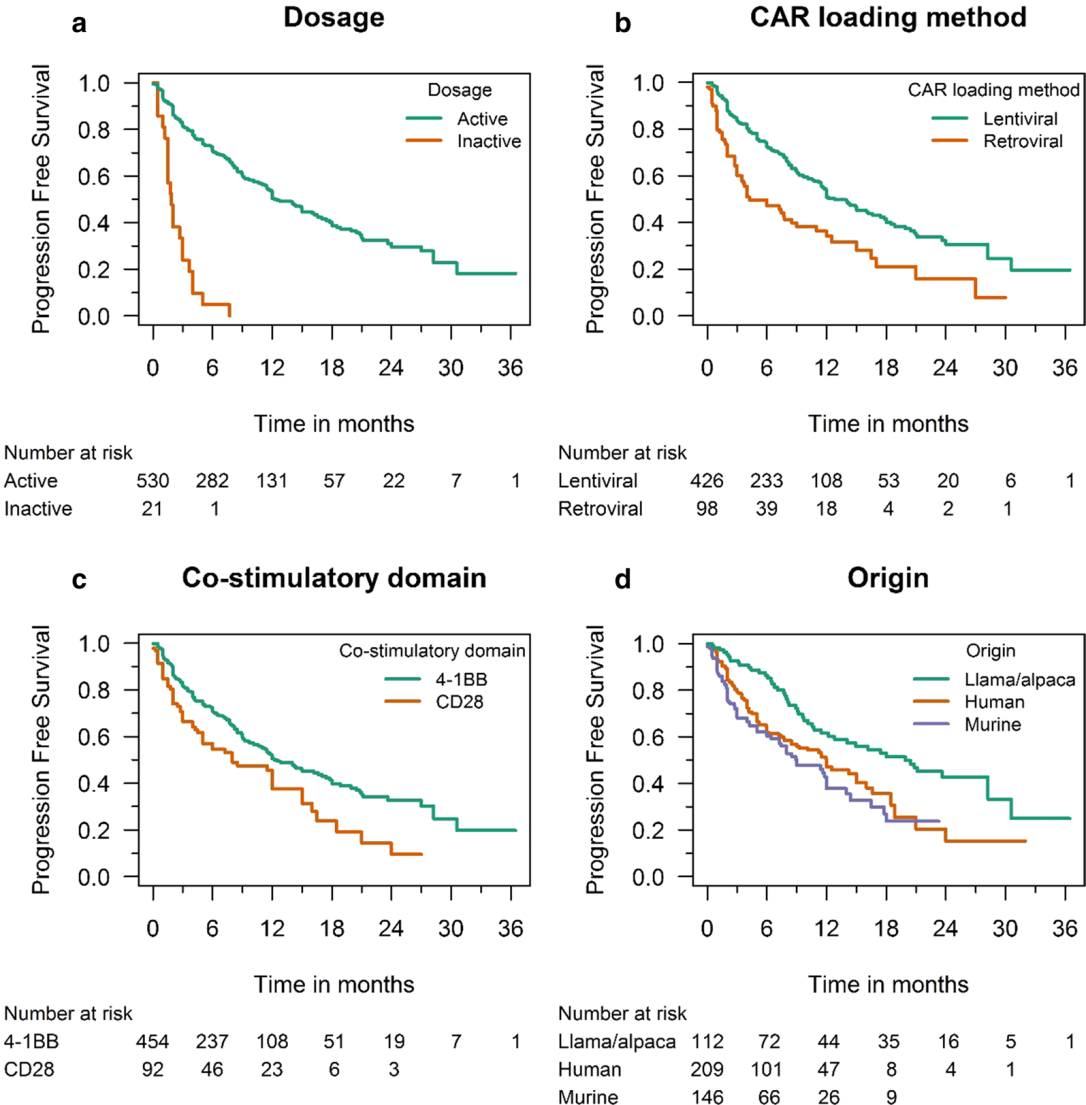
Kaplan–Meier progression-free survival curves

PFS data were available for 551 patients; the median PFS of patients treated with active BCMA CAR-T-cell doses was 12.2 months (11.4–17.4), comparing favorably to the 1.9-month PFS (1.5–3.7) observed in patients treated with inactive doses in the dose-escalation studies NCT02658929, NCT02546167, NCT02215967, and NCT03070327 (hazard ratio [HR] 0.14; *p* < 0.0001; Fig. 3a). In line with the superior clinical response rate, patients treated with lentivirally transduced CAR-T cells had a significantly longer PFS than those treated with retroviral constructs (12.8 months [11.4–19.9] versus 4.3 months [3.0–15.0]; HR 0.48; *p* = 0.0065; Fig. 3b; Additional file 4: Table S4). Although no difference was seen in terms of ORR, we observed a shorter PFS among patients treated with BCMA CAR-T-cells containing a CD28-based co-stimulatory backbone (8.0 months [4.0–15.0] versus 12.2 months [10.8–17.4] with a 4-1BB-based co-stimulatory domain); however, this difference was not statistically significant (HR 0.63, *p* = 0.061; Fig. 3c). The median PFS in the bb2121 study was 12.1 months (8.8–12.3); in the LCAR-B38M study, a median PFS of 19.9 months (9.6–31) was reported (Table 2). The longest PFS rates were observed in studies that used alpaca/llama constructs (*p* = 0.0005; Fig. 3d; Additional file 2: Table S2).

## Discussion

This meta-analysis provides insights into the risk and benefits of BCMA CAR-T-cell therapy in MM, into the diversity of the patient populations included and BCMA CAR-T-cell products used, and into the various factors that potentially contribute to toxicity and efficacy. As of January 1, 2020, 27 registered clinical studies have been published involving 23 different CAR-T-cell products and 640 patients. A high response rate was observed, with demonstrable responses in 8/10 patients (nearly half of whom had a CR). Toxicity was equally high, with CRS occurring in 8/10 patients and neurotoxicity in 1/10 patients. Despite the high initial response rate, responses were usually temporary and relapses were frequently observed, resulting in a median PFS of 12.2 months in patients receiving active doses of BCMA CAR-T cells. The two most advanced BCMA CAR-T products are bb2121 or idecabtagene vicleucel (which contains a murine anti-BCMA scFv as antigen-recognition domain) and LCAR-B38M or ciltacabtagene autoleucel (which contains two llama anti-BCMA heavy chain variable regions or V_H_H) [24, 48, 50].

At the recommended phase II dose level of 450 × 10^6^ cells, the murine BCMA CAR-T product bb2121 yielded comparable CRS rates as its human(ized) or alpaca/llama-based counterparts (Table 2) [59], indicating that not the species origin of the CAR antigen-recognition domain but the CAR-T cell dosage is a major determinant of CRS. Albeit mostly low grade, neurotoxicity occurred in up to 1 out of 5 patients treated with bb2121; in LCAR-B38M-treated patients, the neurotoxicity rate was tenfold lower (Table 2). Since the origin of the antigen-recognition domain (murine, human, or alpaca/llama) was not found to be a risk factor for neurotoxicity in this meta-analysis, other factors should have contributed to the observed difference in neurotoxicity rate between bb2121 and LCAR-B38M. In the LEGEND-2 study conducted at the Xi’An site in China (NCT03090659) [49, 50], the largest study with LCAR-B38M published to date, the lymphodepleting regimen consisted of cyclophosphamide alone, whereas cyclophosphamide and fludarabine were used in the KarMMa pivotal phase II study with bb2121 (NCT03361748) (Table 2). The use of fludarabine for lymphodepletion, which by itself can cause neurotoxicity, was shown to increase the risk of neurologic adverse events in the CD19 CAR-T-cell field [60]. Our meta-analysis, however, failed to demonstrate a role for fludarabine in the higher rate of neurotoxicity in the KarMMa study of bb2121. Dual anti-CD3/CD28 stimulation during CAR-T-cell culture and 4-1BB as co-stimulatory domain were identified as potential risk factors for neurotoxicity in this study. As indicated in Table 2, both KarMMa (bb2121) and LEGEND-2 (LCAR-B38M) used anti-CD3/CD28-stimulated 4-1BB-based CAR-T cells. We, therefore, believe that these factors are not major drivers of neurotoxicity and, at least, do not explain the difference in neurotoxicity rates between both studies. Although this is in sharp contrast with what has been observed in studies with CD19 CAR-T cells [60, 61], this meta-analysis pointed to a higher risk of neurotoxicity in BCMA CAR-T-cell studies in which the median patient age was ≥ 60 years and/or in which the median number of prior lines of anti-myeloma treatments was ≥ 5. As shown in Table 2, LEGEND-2 (LCAR-B38M) tended to include younger and less pretreated patients, possibly explaining the lower frequency of neurological events as compared to KarMMa (bb2121).

Although a previous clinical trial of CD19 CAR-T-cell therapy in CLL failed to demonstrate such correlation [62], we observed a lower rate of deep responses ([s]CR) in studies that included more heavily pretreated patients (≥ 5 prior lines of treatment). This explains why in the LEGEND-2 study a higher proportion of LCAR-B38M-treated patients achieved an (MRD-negative) CR status as compared to the bb2121-treated patients in KarMMA. The rationale behind this is that apheresis products of less pretreated MM patients contain “fitter” T cells [63], resulting in better clinical responses. Autologous BCMA CAR-T-cell therapies are now being positioned earlier in the course of the disease (NCT03549442, NCT03455972) in an attempt to produce deeper and more durable clinical responses. The fact that KarMMa included more high-risk MM patients as compared to LEGEND-2 (Table 2) likely played no role in the lower deep response rate. Indeed, in this meta-analysis, myeloma risk was not associated with reduced activity, indicating that BCMA CAR-T-cell therapy is also highly efficacious in the high need subgroup of high-risk MM patients. Another factor possibly contributing to the superior therapeutic activity of LCAR-B38M is related to the use of (two) llama V_H_Hs as antigen-binding domain in contrast to the murine scFv-based CAR construct of bb2121. It is known that CARs based on heavy-chain-only antibodies (such as alpaca or llama-derived V_H_H) have superior BCMA-binding capability of V_H_H compared to traditional scFv-based domains [64, 65]. This is also reflected by the fact that tenfold lower CAR-T cell dosages were required in LEGEND-2 (LCAR-B38M) as compared to KarMMa (bb2121). To summarize, although head-to-head trials between bb2121 and LCAR-B38M have not been conducted, the results of this meta-analysis indicate that the differences in terms of MRD-negativity, depth of response, and, consequently, PFS, between both products are in large part attributable to the different patient populations included and possibly also to the type of antigen-recognition domain used.

Although there was no statistically significant difference in terms of ORR, PFS was markedly longer in the 4-1BB subgroup. This corroborates recent research showing longer CAR-T-cell persistence and improved response durability with 4-1BB-based as compared to CD28-based CD19 CAR-T cells [66]. Although this should still be confirmed in a randomized controlled trial, our results also seem to favor the use of lentiviral over retroviral vectors for CAR-T-cell transduction given their superior clinical activity without increasing toxicity. Non-viral CAR loading methods, such as DNA transposons, are gaining popularity but how these compare to lentiviral or retroviral transduction in terms of toxicity and activity remains to be established.

We observed a sixfold increase in median PFS in the treatment group compared to the control group, which received an inactive CAR-T-cell dose. The low PFS (~ 2 months) in the control group is congruent with previous literature [67] and illustrates the grim prognosis of the patients included so far in BCMA CAR-T-cell studies. In contrast to what is observed in the field of CD19-directed CAR-T-cell therapy for diffuse large B-cell lymphoma, the tail of the PFS curve did not reveal a plateau. This indicates that the majority of the patients will eventually relapse. Possible explanations are lack of CAR-T cell persistence, antigen escape, the hostile tumor microenvironment, and exhaustion. Persistence can be improved by altering the CD4/CD8 composition of the infusion product [21, 40], or by enriching the product with stem cell memory T cells [45, 68]. BCMA downregulation or loss was observed in several trials [20, 25, 32, 40]; this can be mediated by shedding of BCMA from the cell surface [8] or by CAR-T cell-induced trogocytosis. The latter not only leads to reduced tumor cell recognition, but also to CAR-T-cell fratricide [69]. In order to prevent BCMA shedding, γ-secretase inhibitors are being combined with BCMA CAR-T cells (NCT03502577) [41]. Another approach to circumvent antigen escape is co-targeting of BCMA and another antigen, such as CD19 (NCT03196414, NCT03455972) [27, 43], or simultaneous targeting of two BCMA epitopes as in LCAR-B38M [47–51]. Relapse can also occur despite CAR-T-cell persistence and maintained BCMA expression. The hypoxic niche in the BM, where MM cells reside, impairs cytokine secretion and granzyme B release from BCMA CAR-T cells [70]. In addition, upregulation of immune checkpoint molecules, such as programmed death-1 (PD-1), results in BCMA CAR-T-cell exhaustion which can be restored by PD-1 blockade [71]. Tonic signaling in the absence of antigen can induce CAR-T-cell exhaustion as well; proper selection of the antigen-recognition domain [45] and the co-stimulatory domain [69] can help to minimize CAR tonic signaling.

In conclusion, this meta-analysis provides robust evidence for the high clinical activity of BCMA CAR-T-cell therapies in MM and shows that several patient- and treatment-related factors might contribute to their toxicity and efficacy. These findings may inform the design of future CAR-T-cell studies in MM.

## Supplementary information

**Additional file 1.** Overview of the 61 publications identified following the PRISMA flow diagram.

**Additional file 2.** Subgroup comparison for antigen-recognition domain origin and forest plot for CRS (grouped by antigen-recognition domain).

**Additional file 3.** Subgroup comparison for neurotoxicity and forest plot for neurotoxicity (grouped by lines of prior therapy).

**Additional file 4.** Subgroup comparison for CAR loading method and forest plot for (s)CR (grouped by antigen-recognition domain).

## Data Availability

All data generated or analyzed during this study are included in this published article [and its supplementary information files].
