# Supplementary material for: Safety and clinical efficacy of BCMA CAR-T-cell therapy in multiple myeloma
Source: J Hematol Oncol. 2020 Dec 3;13:164. doi: 10.1186/s13045-020-01001-1 (PMC7713173; doi:10.1186/s13045-020-01001-1)
Supplement: Supplementary file 1 — Additional file 1. Overview of the 61 publications identified following the PRISMA flow diagram. [file 13045_2020_1001_MOESM1_ESM.docx]

**Table S1: Overview of the different publications identified according to the PRISMA flow diagram depicted in Fig. 2.** Publications are ordered based on their reported cut-off date or, if no cut-off date was stated, based on publication year. Reports used for data analysis are in bold. * indicates when the data in the published abstract were combined with the actual data from the oral or poster presentation of this abstract.

| **Trial #**  **(product name)** | **N (cut-off date)** | **Supplementary ref. (SR)** |
| --- | --- | --- |
| ChiCTR-OIC17011272 | **21 (20/01/2019)** | **SR[1]** |
| (CD19 & BCMA CAR-T) |  |  |
| NCT02658929 | 36 (30/04/2018) | SR[2] |
| (bb2121) | **43 (29/03/2018)*** | **SR[3]** |
|  | 21 (02/10/2017) | SR[4] |
|  | 10 (02/10/2017) | SR[5] |
|  | 21 (04/05/2017) | SR[6] |
|  | 11 (18/11/2016) | SR[7] |
|  | 09 (28/10/2016) | SR[8] |
| NCT03274219 | **38 (04/09/2019)*** | **SR[9]** |
| (bb21217) | 8 (15/06/2018) | SR[10] |
| ChiCTR-OPC16009113 | **28 (27/03/2018)** | **SR[11]** |
| (BCMA-CAR T) | 2 | SR[12] |
| NCT02215967 (1) | **10** | **SR[13]** |
| (NCI BCMA CAR-T) | 10 | SR[14] |
|  | 10 | SR[15] |
|  | 6 | SR[16] |
| NCT02215967 (2) | **16** | **SR[13]** |
| (NCI BCMA CAR-T) | 13 | SR[14] |
| ChiCTR-1800018143 | **22 (31/10/2019)*** | **SR[17]** |
| (BM38 CAR) | 12 (31/01/2019) | SR[18] |
| NCT02546167 | 25 (07/09/2018) | SR[19] |
| (CART-BCMA UPenn) | **25 (09/07/2018)** | **SR[20]** |
|  | 21 (24/07/2017) | SR[21] |
|  | 6 | SR[22] |
| NCT03302403, NCT03380039, | **24 (22/09/2018)** | **SR[23]** |
| NCT03716856 | 24 (28/02/2019) | SR[24] |
| (CT053) | 16 (10/07/2018) | SR[25] |
| NCT03430011 | **44** | **SR[26]** |
| (JCARH125) |  |  |
| NCT03815383 | **5 (30/11/2019)*** | **SR[27]** |
| (C-CAR088) |  |  |
| ChiCTR-1800018137 | **18 (28/11/2019)*** | **SR[28]** |
| (CT103A) | 12 (18/06/2019) | SR[29] |
|  | 9 (04/02/2019) | SR[30] |
| NCT03549442 | **16** | **SR[31]** |
| (CART-BCMA+CTL119) |  |  |
| NCT03338972 | **11*** | **SR[32]** |
| (FCARH143) |  |  |
| NCT03502577 | **11*** | **SR[33]** |
| (FCARH143+GSI) |  |  |
| NCT03196414 | **28 (31/01/2019)** | **SR[34]** |
| (SZ-MM-CART01) | 8 | SR[35] |
| NCT03455972 | **32** | **SR[36]** |
| (SZ-MM-CART02) | 10 | SR[37] |
|  | 9 | SR[38] |
| NCT03070327 | **11 (16/07/2018)** | **SR[39]** |
| (MCARH171) | 6 | SR[40] |
| NCT03602612 | **15*** | **SR[41]** |
| (FHVH33) |  |  |
| NCT03288493 | **23 (29/11/2018)*** | **SR[42]** |
| (P-BCMA-101) | 2 | SR[43] |
| NCT03661554 | 16 (31/12/2018) | SR[44] |
| (BCMA nanoantibody) | **9*** | **SR[45]** |
| NCT03090659 (1) | **17 (20/07/2019)** | **SR[46]** |
| (LCAR-B38M) | 17 (20/10/2018) | SR[47] |
| NCT03090659 (2) | **57 (31/12/2018)** | **SR[48]** |
| (LCAR-B38M) | 57 (25/06/2018) | SR[49] |
|  | 57 (06/02/2018) | SR[50] |
|  | 19 | SR[51] |
|  | 5 (31/07/2017) | SR[52] |
|  | 22 (20/02/2017) | SR[53] |
| NCT03549207 | **29*** | **SR[54]** |
| (LCAR-B38M) |  |  |
| ChiCTR-1800017404 | **33 (01/08/2019)** | **SR[55]** |
| (BCMA CAR-T) | 19 (01/12/2018) | SR[56] |
|  | 17 (28/11/2018) | SR[57] |
| NCT03093168 | **49*** | **SR[58]** |
| (HRAIN BCMA-CART) | 17 (06/07/2018) | SR[59] |
|  | 10 (31/12/2017) | SR[60] |
| ChiCTR-1900027678 | **5 (31/07/2019)** | **SR[61]** |
| (GC012F) |  |  |

**Supplementary references**

SR[1] Yan Z, Cao J, Cheng H, Qiao J, Zhang H, Wang Y, et al. A combination of humanised anti-CD19 and anti-BCMA CAR T cells in patients with relapsed or refractory multiple myeloma: a single-arm, phase 2 trial. Lancet Haematol. 2019;6(10):e521-e9.

SR[2] Raje N, Berdeja J, Lin Y, Siegel D, Jagannath S, Madduri D, et al. Anti-BCMA CAR T-cell therapy bb2121 in relapsed or refractory multiple myeloma. N Engl J Med. 2019;380(18):1726-37.

SR[3] Raje NS, Berdeja JG, Lin Y, Munshi NC, Siegel DSD, Liedtke M, et al. bb2121 anti-BCMA CAR T-cell therapy in patients with relapsed/refractory multiple myeloma: updated results from a multicenter phase I study. J Clin Oncol. 2018;36:8007.

SR[4] Einsele H, Goldschmidt H, Weisel K, Hege K, Munshi N, San Miguel J. Preliminary results of a phase I trial inspire the phase II KarMMa study of bb2121, an anti-B cell maturation antigen (BCMA) chimeric antigen receptor (CAR) T cell therapy, for relapsed and refractory multiple myeloma (rrMM). Oncol Res Treat. 2018;41:134.

SR[5] Munshi NC, Berdeja JG, Lin Y, Kochenderfer J, Raje NS, Liedtke M, et al. Early MRD negativity to predict deepening myeloma response in relapsed/refractory multiple myeloma (RRMM) patients treated with bb2121 anti-BCMA CAR T cells. J Clin Oncol. 2018;36:8024.

SR[6] Berdeja JG, Lin Y, Raje N, Munshi N, Siegel D, Liedtke M, et al. Durable clinical responses in heavily pretreated patients with relapsed/refractory multiple myeloma: updated results from a multicenter study of bb2121 anti-BCMA CAR T cell therapy. Blood. 2017;130:740.

SR[7] Berdeja JG, Lin Y, Raje NS, Siegel DSD, Munshi NC, Liedtke M, et al. First-in-human multicenter study of bb2121 anti-BCMA CAR T-cell therapy for relapsed/refractory multiple myeloma: updated results. J Clin Oncol. 2017;35:3010.

SR[8] Berdeja JG, Lin Y, Raje N, Siegel D, Munshi N, Turka A, et al. Clinical remissions and limited toxicity in a first-in-human multicenter study of bb2121, a novel anti-BCMA CAR T cell therapy for relapsed/refractory multiple myeloma. Eur J Cancer. 2016;69:S5.

SR[9] Berdeja JG, Alsina M, Shah ND, Siegel DS, Jagannath S, Madduri D, et al. Updated results from an ongoing phase 1 clinical study of bb21217 anti-BCMA CAR-T-cell therapy. Blood. 2019;134:927.

SR[10] Shah N, Alsina M, Siegel DS, Jagannath S, Madduri D, Kaufman JL, et al. Initial results from a phase 1 clinical study of bb21217, a next-generation anti bcma CAR T therapy. Blood. 2018;132:488.

SR[11] Li C, Wang Q, Zhu H, Mao X, Wang Y, Zhang Y, et al. T cells expressing anti B-cell maturation antigen chimeric antigen receptors for plasma cell malignancies. Blood. 2018;132:1013.

SR[12] Xu J, Wang Q, Xu H, Gu C, Jiang L, Wang J, et al. Anti-BCMA CAR-T cells for treatment of plasma cell dyscrasia: case report on POEMS syndrome and multiple myeloma. J Hematol Oncol. 2018;11(1):128.

SR[13] Brudno JN, Maric I, Hartman SD, Rose JJ, Wang M, Lam N, et al. T cells genetically modified to express an anti-B-cell maturation antigen chimeric antigen receptor cause remissions of poor-prognosis relapsed multiple myeloma. J Clin Oncol. 2018;36(22):2267-80.

SR[14] Brudno J, Lam N, Wang M, Stroncek D, Maric I, Stetler-Stevenson M, et al. T cells genetically modified to express an anti-B-cell maturation antigen chimeric antigen receptor with a CD28 costimulatory moiety cause remissions of poor-prognosis relapsed multiple myeloma. Blood. 2017;130:524.

SR[15] Ali SA, Shi V, Maric I, Wang M, Stroncek DF, Rose JJ, et al. T cells expressing an anti–B-cell maturation antigen chimeric antigen receptor cause remissions of multiple myeloma. Blood. 2016;128(13):1688-700.

SR[16] Ali SA, Shi V, Wang M, Stroncek D, Maric I, Brudno JN, et al. Remissions of multiple myeloma during a first-in-humans clinical trial of T cells expressing an anti-B-cell maturation antigen chimeric antigen receptor. Blood. 2015;126(23).

SR[17] Li C, Mei H, Hu Y, Guo T, Liu L, Jiang H, et al. A bispecific CAR-T cell therapy targeting BCMA and CD38 for relapsed/refractory multiple myeloma: updated results from a phase 1 dose-climbing trial. Blood. 2019;134:930.

SR[18] Li C, Mei H, Hu Y, Guo T, Liu L, Jiang H, et al. Improved efficacy and safety of a dual-target CAR-T cell therapy targeting BCMA and CD38 for relapsed/refractory multiple myeloma from a phase I study. Eur J Immunol. 2019;49:1723-4.

SR[19] Cohen AD, Melenhorst JJ, Garfall AL, Lacey SF, Davis M, Vogl DT, et al. Predictors of T cell expansion and clinical responses following B-cell maturation antigen-specific chimeric antigen receptor T cell therapy (CART-BCMA) for relapsed/refractory multiple myeloma (MM). Blood. 2018;132:1974.

SR[20] Cohen AD, Garfall AL, Stadtmauer EA, Melenhorst JJ, Lacey SF, Lancaster E, et al. B cell maturation antigen–specific CAR T cells are clinically active in multiple myeloma. J Clin Invest. 2019;129(6):2210-21.

SR[21] Cohen AD, Garfall AL, Stadtmauer EA, Lacey SF, Lancaster E, Vogl DT, et al. Safety and efficacy of B-cell maturation antigen (BCMA)-specific chimeric antigen receptor T cells (CART-BCMA) with cyclophosphamide conditioning for refractory multiple myeloma (MM). Blood. 2017;130:505.

SR[22] Cohen AD, Garfall AL, Stadtmauer EA, Lacey SF, Lancaster E, Vogl DT, et al. B-cell maturation antigen (BCMA)-specific chimeric antigen receptor T cells (CART-BCMA) for multiple myeloma (MM): initial safety and efficacy from a phase I study. Blood. 2016;128(22):1147.

SR[23] Jie J, Hao S, Jiang S, Li Z, Yang M, Zhang W, et al. Phase 1 trial of the safety and efficacy of fully human anti-bcma CAR T cells in relapsed/refractory multiple myeloma. Blood. 2019;134:4435.

SR[24] Hao S, Jin J, Yu K, Li Z, Zhang W, Yang M, et al. CT053, anti-BCMA CAR T-cell therapy for relapsed/refractory multiple myeloma: proof of concept results from a phase I study. Clin Lymphoma Myeloma Leuk. 2019;19:e54-e5.

SR[25] Jiang S, Jin J, Hao S, Yang M, Chen L, Ruan H, et al. Low dose of human scFv-derived BCMA-targeted CAR-T cells achieved fast response and high complete remission in patients with relapsed/refractory multiple myeloma. Blood. 2018;132:960.

SR[26] Mailankody S, Htut M, Lee KP, Bensinger W, Devries T, Piasecki J, et al. JCARH125, anti-BCMA CAR T-cell therapy for relapsed/refractory multiple myeloma: initial proof of concept results from a phase 1/2 multicenter study (EVOLVE). Blood. 2018;132:957.

SR[27] Yao X, Zhu S, Huang J, Qu X, Zhu J, Wei Y, et al. Developing a novel anti-BCMA CAR-T for relapsed or refractory multiple myeloma. Blood. 2019;134:50.

SR[28] Li C, Wang J, Wang D, Hu G, Yang Y, Zhou X, et al. Efficacy and safety of fully human bcma targeting CAR T cell therapy in relapsed/refractory multiple myeloma. Blood. 2019;134:929.

SR[29] Li C, Zhou X, Wang J, Hu G, yang y, Meng L, et al. Clinical responses and pharmacokinetics of fully human BCMA targeting CAR T-cell therapy in relapsed/refractory multiple myeloma. Clin Lymphoma Myeloma Leuk. 2019;19:e23-e4.

SR[30] Li C, Zhou J, Wang J, Hu G, Du A, Zhou X, et al. Clinical responses and pharmacokinetics of fully human BCMA targeting CAR T-cell therapy in relapsed/refractory multiple myeloma. J Clin Oncol. 2019;37:8013.

SR[31] Garfall AL, Cohen AD, Lacey SF, Tian L, Hwang W-T, Vogl DT, et al. Combination anti-BCMA and anti-CD19 CAR T cells as consolidation of response to prior therapy in multiple myeloma. Blood. 2019;134:186-.

SR[32] Green DJ, Pont M, Sather BD, Cowan AJ, Turtle CJ, Till BG, et al. Fully human BCMA targeted chimeric antigen receptor T cells administered in a defined composition demonstrate potency at low doses in advanced stage high risk multiple myeloma. Blood. 2018;132:1011.

SR[33] Cowan AJ, Pont M, Sather BD, Turtle CJ, Till BG, Nagengast AM, et al. Efficacy and safety of fully human bcma CAR T cells in combination with a gamma secretase inhibitor to increase bcma surface expression in patients with relapsed or refractory multiple myeloma. Blood. 2019;134:204.

SR[34] Yan L, Yan Z, Shang J, Shi X, Jin S, Kang L, et al. Sequential CD19- and BCMA-specific chimeric antigen receptor T cell treatment for RRMM: report from a single center study. Blood. 2019;134:578.

SR[35] Yan L, Shang J, Kang L, Shi X, Zhou J, Jin S, et al. Combined infusion of CD19 and bcma-specific chimeric antigen receptor T cells for RRMM: initial safety and efficacy report from a clinical pilot study. Blood. 2017;130:506.

SR[36] Shi X, Yan L, Shang J, Kang L, Jin S, Kang H, et al. Combined infusion of anti-CD19 and anti-BCMA CART cells after early or later transplantation in the front line was superior to salvage therapy for high risk MM. Blood. 2019;134:1949.

SR[37] Shi X, Yan L, Shang J, Kang L, Qu S, Sun J, et al. Tandom autologous transplantation and combined infusion of CD19 and BCMA-specific chimeric antigen receptor T cells for high risk MM. 45th Annual Meeting of the European Society for Blood and Marrow Transplantation. 2019.

SR[38] Shi X, Yan L, Shang J, Qu S, Kang L, Zhou J, et al. Tandom autologous transplantation and combined infusion of CD19 and BCMA-specific chimeric antigen receptor T cells for high risk MM: initial safety and efficacy report from a clinical pilot study. Blood. 2018;132:1009.

SR[39] Mailankody S, Ghosh A, Staehr M, Purdon TJ, Roshal M, Halton E, et al. Clinical responses and pharmacokinetics of MCARH171, a human-derived BCMA targeted CAR T cell therapy in relapsed/refractory multiple myeloma: final results of a phase I clinical trial. Blood. 2018;132:959.

SR[40] Smith EL, Mailankody S, Ghosh A, Masakayan R, Staehr M, Purdon TJ, et al. Development and evaluation of a human single chain variable fragment (scFv) derived BCMA targeted CAR T cell vector leads to a high objective response rate in patients with advanced MM. Blood. 2017;130:742.

SR[41] Mikkilineni L, Manasanch EE, Lam N, Vanasse D, Brudno JN, Maric I, et al. T cells expressing an anti-B-cell maturation antigen (BCMA) chimeric antigen receptor with a fully-human heavy-chain-only antigen recognition domain induce remissions in patients with relapsed multiple myeloma. Blood. 2019;134:3230.

SR[42] Gregory T, Cohen AD, Costello CL, Ali SA, Berdeja JG, Ostertag EM, et al. Efficacy and safety of p-BCMA-101 CAR-T cells in patients with relapsed/refractory (r/r) multiple myeloma (MM). Blood. 2018;132:1012.

SR[43] Gregory TK, Berdeja JG, Patel KK, Ali SA, Cohen AD, Costello C, et al. Clinical trial of P-BCMA-101 T stem cell memory (Tscm) CAR-T cells in relapsed/refractory (r/r) multiple myeloma (MM). Cancer Res. 2018;78.

SR[44] Han L, Gao Q, Zhou K, Zhou J, Fang B, Zhang J, et al. The phase I clinical study of CART targeting BCMA with humanized alpaca-derived single-domain antibody as antigen recognition domain. J Clin Oncol. 2019;37:2535.

SR[45] Han L, Gao Q, Zhou K, Yin Q, Fang B, Zhou J, et al. Development and evaluation of CART targeting BCMA with humanized alpaca-derived single-domain antibody as antigen recognition domain. Blood. 2018;132:1976.

SR[46] Chen L, Xu J, Fu W, Sr., Jin S, Yang S, Yan S, et al. Updated phase 1 results of a first-in-human open-label study of LCAR-B38M, a structurally differentiated chimeric antigen receptor T (CAR-T) cell therapy targeting B-cell maturation antigen (BCMA). Blood. 2019;134:1858.

SR[47] Xu J, Chen L-J, Yang S-S, Sun Y, Wu W, Liu Y-F, et al. Exploratory trial of a biepitopic CAR T-targeting B cell maturation antigen in relapsed/refractory multiple myeloma. Proc Natl Acad Sci U S A. 2019;116(19):9543.

SR[48] Wang B-Y, Zhao W-H, Liu J, Chen Y-X, Cao X-M, Yang Y, et al. Long-term follow-up of a phase 1, first-in-human open-label study of LCAR-B38M, a structurally differentiated chimeric antigen receptor T (CAR-T) cell therapy targeting B-cell maturation antigen (BCMA), in patients (pts) with relapsed/refractory multiple myeloma (RRMM). Blood. 2019;134:579.

SR[49] Zhao W-H, Liu J, Wang B-Y, Chen Y-X, Cao X-M, Yang Y, et al. Updated analysis of a phase 1, open-label study of LCAR-B38M, a chimeric antigen receptor T cell therapy directed against B-cell maturation antigen, in patients with relapsed/refractory multiple myeloma. Blood. 2018;132:955.

SR[50] Zhao W-H, Liu J, Wang B-Y, Chen Y-X, Cao X-M, Yang Y, et al. A phase 1, open-label study of LCAR-B38M, a chimeric antigen receptor T cell therapy directed against B cell maturation antigen, in patients with relapsed or refractory multiple myeloma. J Hematol Oncol. 2018;11(1):141.

SR[51] Fan F, Zhao W, Liu J, He A, Chen Y, Cao X, et al. Durable remissions with BCMA-specific chimeric antigen receptor (CAR)-modified T cells in patients with refractory/relapsed multiple myeloma. J Clin Oncol. 2017;35.

SR[52] Mi J-Q, Fan X, Xu J, Liu Y, Zhuang Y, Yang S, et al. Effective treatment of relapsed/refractory multiple myeloma including extramedullary involvement by BCMA-specific chimeric antigen receptor-modified T cells. Blood. 2017;130:3115.

SR[53] Zhang W, Zhao W, Liu J, He A, Chen Y, Cao X, et al. Phase I, open-label trial of anti-BCMA chimeric antigen receptor T cells in patients with relapsed/refractory multiple myeloma. Haematologica. 2017;102:2-3.

SR[54] Madduri D, Usmani SZ, Jagannath S, Singh I, Zudaire E, Yeh T-M, et al. Results from CARTITUDE-1: a phase 1b/2 study of JNJ-4528, a CAR-T cell therapy directed against B-cell maturation antigen (BCMA), in patients with relapsed and/or refractory multiple myeloma (R/R MM). Blood. 2019;134:577.

SR[55] Hu Y, Yanlei Z, Wei G, alex Hong C, Huang H. Potent anti-tumor activity of BCMA CAR-T therapy against heavily treated multiple myeloma and dynamics of immune cell subsets using single-cell mass cytometry. Blood. 2019;134:1859.

SR[56] Shao M, Hu Y, Xu H, Zhang Y, Cui J, Huang H. Coagulation disorders during CRS of a BCMA targeted CAR T cell therapy in relapsed/refractory multiple myeloma: periodic results of a phase I clinical trial. 45th Annual Meeting of the European Society for Blood and Marrow Transplantation 2019.

SR[57] Hu Y, Zhang Y, Wei G, Xu H, Wu W, Liu S, et al. High expansion level and long term persistence of BCMA CAR-T cells contribute to the potent anti-tumor activity against heavily treated multiple myeloma patients. 45th Annual Meeting of the European Society for Blood and Marrow Transplantation 2019.

SR[58] Fu W, Sr., Du J, Jiang H, Cheng Z, Wei R, Yu K, et al. Efficacy and safety of CAR-T therapy with safety switch targeting bcma for patients with relapsed/refractory multiple myeloma in a phase 1 clinical study. Blood. 2019;134:3154.

SR[59] Liu Y, Chen Z, Fang H, Wei R, Yu K, Jiang S, et al. Durable remission achieved from BCMA-directed CAR-T therapy against relapsed or refractory multiple myeloma. Blood. 2018;132:956.

SR[60] Liu Y, Chen Z, Wei R, Shi L, He F, Shi Z, et al. Remission observed from a phase 1 clinical study of CAR-T therapy with safety switch targeting BCMA for patients with relapsed/refractory multiple myeloma. J Clin Oncol. 2018;36:8020.

SR[61] Zhang H, Gao L, Liu L, Wang J, Wang S, Gao L, et al. A BCMA and CD19 bispecific CAR-T for relapsed and refractory multiple myeloma. Blood. 2019;134:3147.
