# Supplementary material for: Safety and clinical efficacy of BCMA CAR-T-cell therapy in multiple myeloma
Source: J Hematol Oncol. 2020 Dec 3;13:164. doi: 10.1186/s13045-020-01001-1 (PMC7713173; doi:10.1186/s13045-020-01001-1)

**Table S2: Subgroup comparison for antigen-recognition domain origin.**

|  | **Human** | **Murine** | **Llama/alpaca** |  |
| --- | --- | --- | --- | --- |
|  | **Proportion** | **Proportion** | **Proportion** |  |
|  | **(95% CI)** | **(95% CI)** | **(95% CI)** | **P-value** |
| **Number of studies** | 16 | 5 | 3 |  |
| **Number of patients** | 285 | 156 | 112 |  |
| **CRS grade 1-2** | 68.24% | 53.30% | 76.26% | 0.15 |
|  | (56.59 – 77.98%) | (34.24 – 71.45%) | (61.88 – 86.41%) |  |
| **CRS grade 3-4** | 15.58% | 10.11% | 15.74% | 0.67 |
|  | (9.66 – 24.18%) | (4.08 - 22.93%) | (5.26 - 38.59%) |  |
| **CRS all grades** | 83.83% | 61.03% | 91.00% | 0.013 |
|  | (70.90 – 91.69%) | (35.32 – 81.79%) | (83.83 – 95.18%) |  |
| **Neurotoxicity** | 12.63% | 14.88% | 6.48% | 0.31 |
|  | (6.96 – 21.85%) | (7.00 – 28.87%) | (2.69 – 14.79%) |  |
| **CR** | 39.88% | 31.97% | 71.91% | < 0.0001 |
|  | (26.69 – 54.73%) | (16.02 – 53.66%) | (62.76 – 79.55%) |  |
| **CR/VGPR** | 56.40% | 59.70% | 81.53% | 0.0013 |
|  | (43.12 – 68.83%) | (44.92 – 72.90%) | (73.04 – 87.79%) |  |
| **ORR** | 78.72% | 72.24% | 89.52% | 0.060 |
|  | (69.32 – 85.83%) | (50.95 – 86.70%) | (81.57 – 94.29) |  |
| **Median PFS** | 12.0m | 9.0m | 19.9m | 0.0005 |
|  | (7.4 – 16.6) | (5.0 – 14.4) | (16.2 – 28.2) |  |

**Fig. S1: Forest plot for CRS, grouped by antigen-recognition domain origin.**


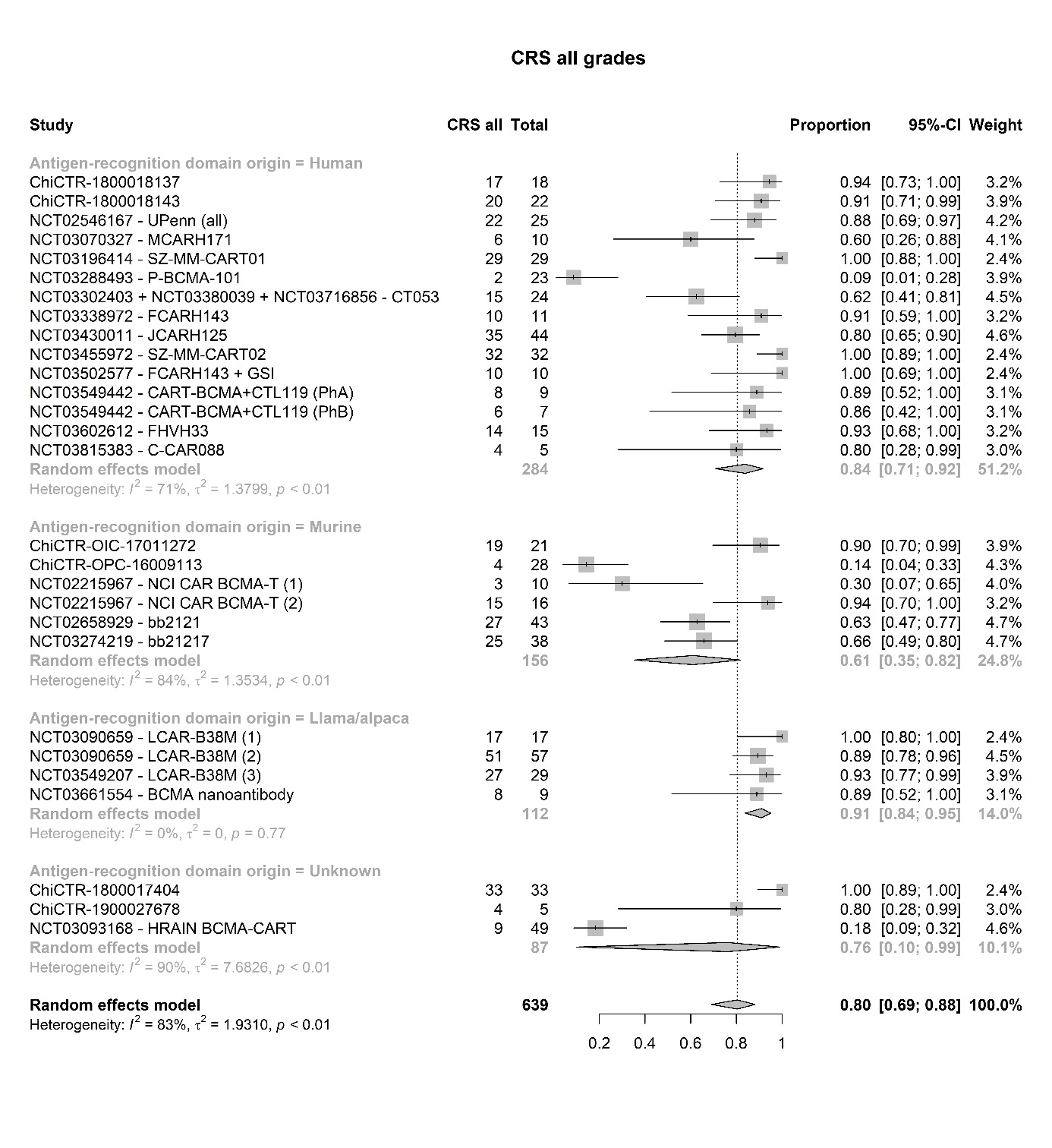

Supplement: Supplementary file 2 — Additional file 2. Subgroup comparison for antigen-recognition domain origin and forest plot for CRS (grouped by antigen-recognition domain). [file 13045_2020_1001_MOESM2_ESM.docx]
