# Supplementary material for: Safety and clinical efficacy of BCMA CAR-T-cell therapy in multiple myeloma
Source: J Hematol Oncol. 2020 Dec 3;13:164. doi: 10.1186/s13045-020-01001-1 (PMC7713173; doi:10.1186/s13045-020-01001-1)

**Table S3: Subgroup comparison for neurotoxicity.**

|  | **Condition = FALSE** | | **Condition = TRUE** | |  |
| --- | --- | --- | --- | --- | --- |
| **Condition** | **N studies** | **Proportion** | **N studies** | **Proportion** | **P-value** |
|  | **(patients)** | **neurotoxicity** | **(patients)** | **neurotoxicity** |  |
|  |  | **(95% CI)** |  | **(95%CI)** |  |
| **Median age ≥ 60** | 16 (326) | 6.41% | 9 (259) | 20.51% | 0.0043 |
|  |  | (3.33% – 12.00%) |  | (12.47% – 31.86%) |  |
| **≥ 50% high risk** | 11 (321) | 9.35% | 13 (243) | 13.58% | 0.42 |
|  |  | (4.74% – 17.61%) |  | (7.10% – 24.42%) |  |
| **≥ 5 prior lines of** | 10 (245) | 2.78% | 16 (333) | 19.10% | < 0.0001 |
| **treatment** |  | (1.25% – 6.06%) |  | (13.30% – 26.66%) |  |
| **Recognition domain** | 21 (440) | 13.76% | 4 (112) | 6.48% | 0.12 |
| **= Llama/alpaca** |  | (8.84 – 20.80%) |  | (2.69 – 14.79%) |  |
| **Enrichment/activation** | 6 (136) | 4.92% | 7 (215) | 15.88% | 0.028 |
| **= aCD3/CD28** |  | (2.14% – 10.90%) |  | (8.07% – 28.89%) |  |
| **Loading method** | 5 (100) | 8.73% | 20 (489) | 11.86% | 0.62 |
| **= lentiviral** |  | (2.75% – 24.44%) |  | (7.22% – 18.90%) |  |
| **Co-stimulation** | 5 (115) | 3.41% | 22 (519) | 12.85% | 0.018 |
| **= 4-1BB** |  | (1.20% – 9.31%) |  | (8.21% – 19.56%) |  |
| **Lymphodepletion** | 6 (138) | 7.27% | 23 (501) | 11.28% | 0.53 |
| **= CP/Flu** |  | (1.89% – 24.25%) |  | (7.10% – 17.46%) |  |

**Fig. S2: Forest plot for neurotoxicity, grouped by lines of prior therapy.**


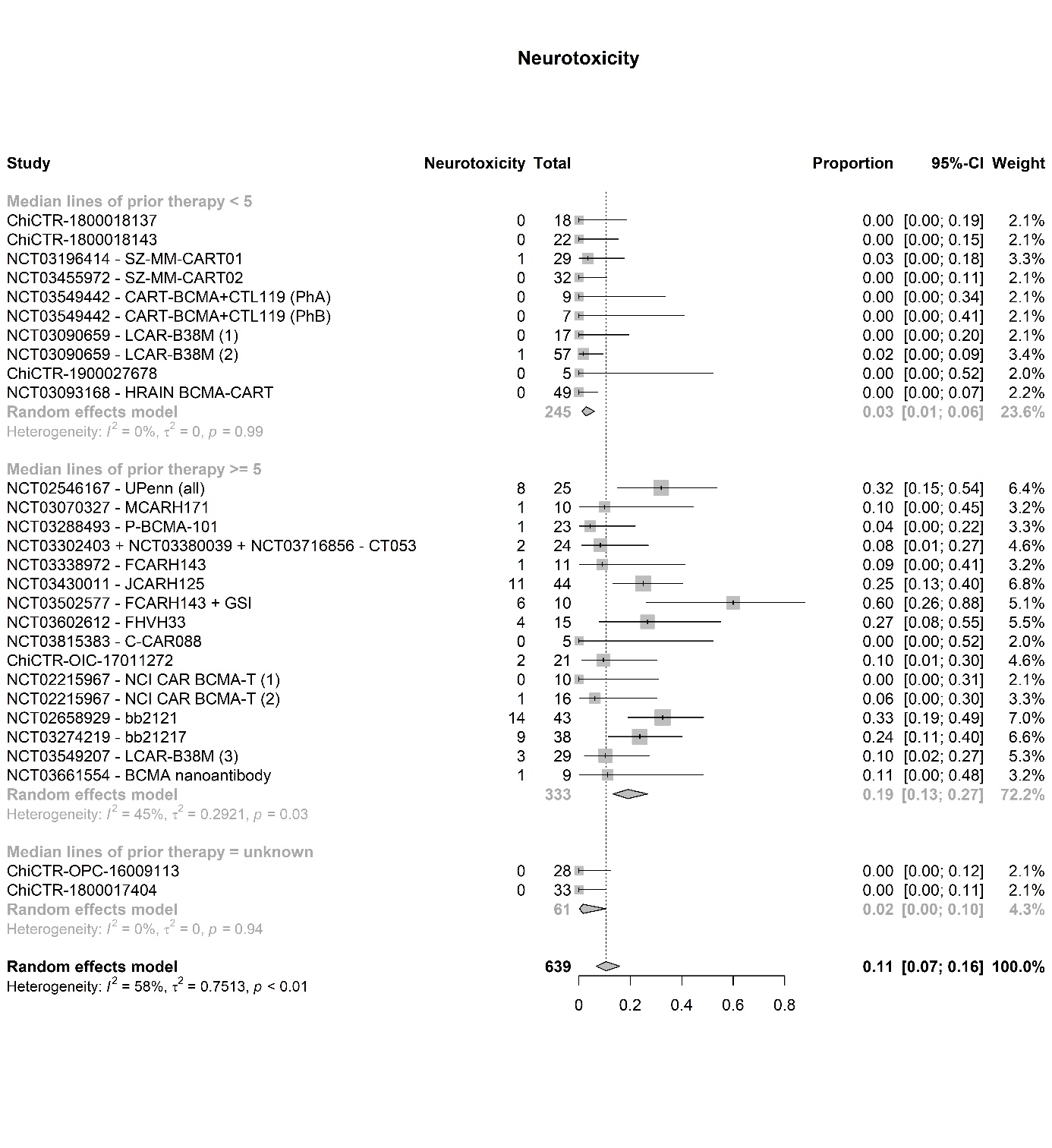

Supplement: Supplementary file 3 — Additional file 3. Subgroup comparison for neurotoxicity and forest plot for neurotoxicity (grouped by lines of prior therapy). [file 13045_2020_1001_MOESM3_ESM.docx]
