# Supplementary material for: Safety and clinical efficacy of BCMA CAR-T-cell therapy in multiple myeloma
Source: J Hematol Oncol. 2020 Dec 3;13:164. doi: 10.1186/s13045-020-01001-1 (PMC7713173; doi:10.1186/s13045-020-01001-1)

**Table S4: Subgroup comparison for CAR loading method.**

|  | **Retroviral** | **Lentiviral** |  |
| --- | --- | --- | --- |
|  | **Proportion** | **Proportion** |  |
|  | **(95% CI)** | **(95% CI)** | **P-value** |
| **Number of studies** | 4 | 20 |  |
| **Number of patients** | 101 | 489 |  |
| **CRS grade 1-2** | 43.05% | 69.63% | 0.094 |
|  | (17.82% – 72.49%) | (61.62% – 76.61%) |  |
| **CRS grade 3-4** | 13.43% | 14.32% | 0.90 |
|  | (4.92% – 31.75%) | (8.91% – 22.22%) |  |
| **CRS all grades** | 62.23% | 85.02% | 0.16 |
|  | (25.03% – 89.05%) | (75.61% – 91.22%) |  |
| **Neurotoxicity** | 8.73% | 11.86% | 0.62 |
|  | (2.75% – 24.44%) | (7.22% – 18.90%) |  |
| **CR** | 18.01% | 50.62% | 0.015 |
|  | (6.48% – 41.06%) | (39.77% – 61.42%) |  |
| **CR/VGPR** | 44.92% | 70.51% | 0.022 |
|  | (26.56% – 64.78%) | (60.87% – 78.61%) |  |
| **ORR** | 68.33% | 84.14% | 0.076 |
|  | (48.07% – 83.42%) | (75.96% – 89.91%) |  |
| **Median PFS** | 4.3m | 12.8m | 0.0065 |
|  | (3.0 – 15.0) | (11.4 – 19.9) |  |

**Fig. S3: Forest plot for (s)CR, grouped by antigen-recognition domain origin.**


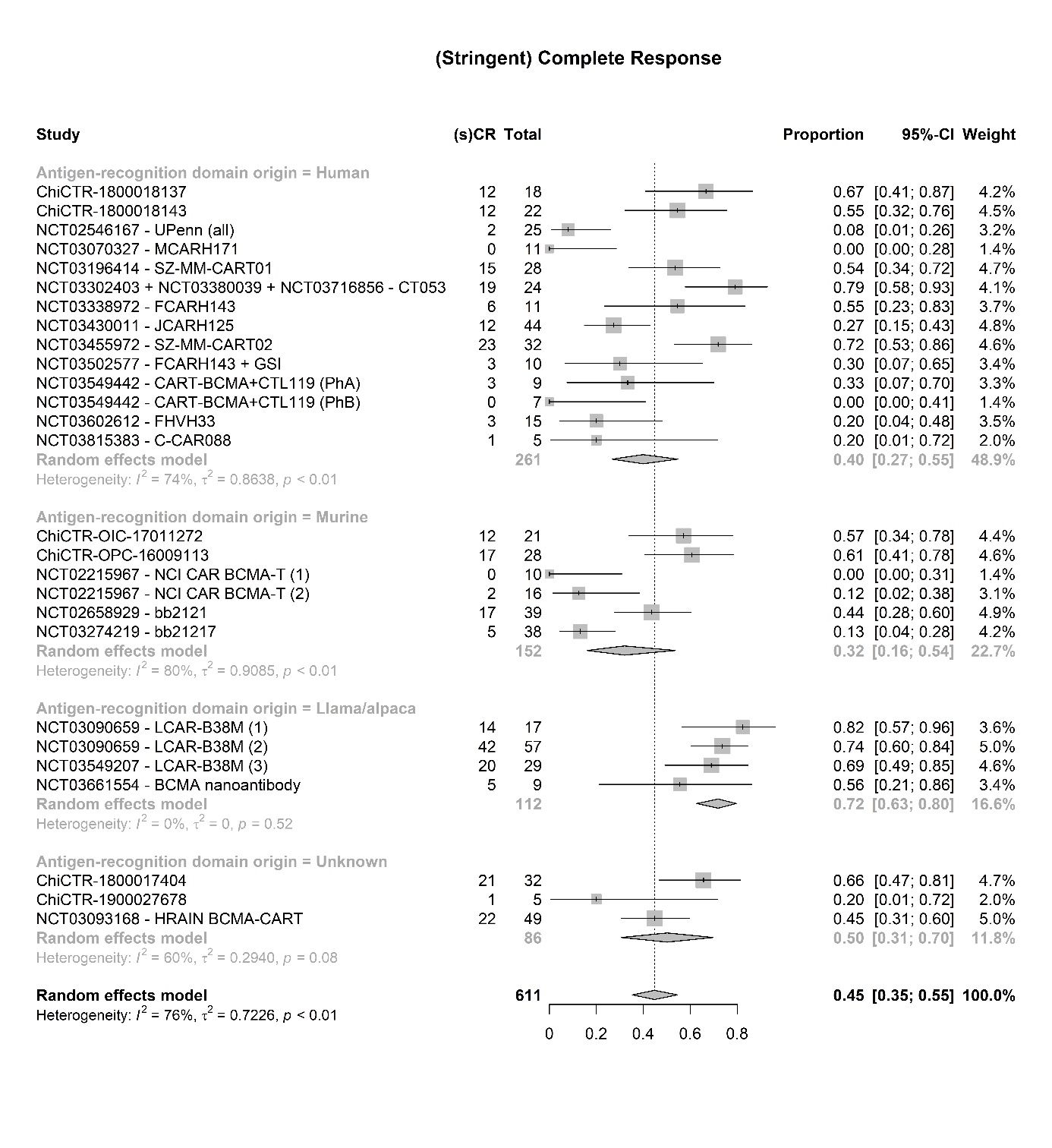

Supplement: Supplementary file 4 — Additional file 4. Subgroup comparison for CAR loading method and forest plot for (s)CR (grouped by antigen-recognition domain). [file 13045_2020_1001_MOESM4_ESM.docx]
